# Socio-Economic Disparities in Early Childhood Education Enrollment: Japanese Population-Based Study

**DOI:** 10.2188/jea.JE20180216

**Published:** 2020-03-05

**Authors:** Yuko Kachi, Tsuguhiko Kato, Ichiro Kawachi

**Affiliations:** 1 Department of Public Health, Kitasato University School of Medicine, Kanagawa, Japan; 2 Department of Social Medicine, National Center for Child Health and Development, Tokyo, Japan; 3 Department of Social and Behavioral Sciences, Harvard T.H. Chan School of Public Health, Boston, MA, USA

**Keywords:** early childhood education and care, socio-economic status, children with special health and developmental needs, Japanese family and children

## Abstract

**Background:**

Accumulating evidence has shown that high-quality early childhood education and care may be an effective way of promoting children’s optimal health and development, especially for the most disadvantaged. However, socially disadvantaged families are less likely to enroll children in center-based childcare. In this study, we explored characteristics associated with use of center-based childcare among Japanese families.

**Methods:**

We used data from two Japanese birth cohorts in 2001 (*n* = 17,019) and 2010 (*n* = 24,333). Enrollment in center-based childcare was assessed at the ages of three and four years in the 2001 cohort and at the age of three in the 2010 cohort. Logistic regression analyses were conducted.

**Results:**

Children in the lowest quintile of household income were 1.54 (95% confidence interval, 1.20–1.98) times more likely to not receive center-based childcare than those in the highest-income quartile at the age of four in the 2001 cohort. Other socio-economic disadvantage (mother’s low education, non-Japanese parent, and higher number of siblings) and child’s health and developmental problems (preterm birth, congenital diseases, and developmental delay) were also associated with the non-use of center-based childcare at the age of three in the 2001 and 2010 cohorts.

**Conclusions:**

An inverse care law operates in the use of early childhood education (ie, children with the least need enjoy the highest access). Children with socio-economic, health, and developmental disadvantages are at a greater risk of not receiving early childhood education and care. Social policies to promote equal access to early childhood education are needed to reduce future socio-economic inequalities.

## INTRODUCTION

Accumulating evidence has shown that high-quality early childhood education and care (ECEC) may be an effective and efficient way of promoting children’s optimal health and development, both in the short term and long term.^1^^–^^4^ Recent studies also have shown that the benefits of ECEC may be most pronounced among socioeconomically disadvantaged children.^5^^,^^6^ In Japan, Yamaguchi et al found that enrollment in center-based childcare may lower the risk of behavior problems, such as hyperactivity, among children of mothers with high school education or less and enhance parenting quality among the low-education mothers.^7^

However, previous studies conducted in developed countries have shown that access to ECEC may not be equal among families; namely, children from socially disadvantaged families may be more likely to end up in parental childcare over non-parental childcare, such as center-based childcare, despite the benefits of enrolling children in ECEC.^8^ For example, in the United States, one study using a national database showed that family needs and resources, cultural norms and preferences, and contextual opportunities and constraints were all associated with the selection of ECEC programs.^9^ Another American study found that maternal employment and education predicted the selection of ECEC among low-income families.^10^ An Australian study showed that disadvantages, such as non-English speaking and poverty, were associated with non-enrollment in preschool.^11^ A Canadian study also found that factors, such as younger maternal age and higher number of children, were associated with a higher chance of not receiving ECEC.^12^ Sylva et al found that socio-economic disadvantage was associated with lower chance of non-parental care for infants in England.^13^ Zachrisson et al found that parental preference, being immigrants, and lower socio-economic status were associated with lower chance of receiving ECEC prior to the child’s age of 18 months in Norway.^14^

A recent report comparing expenditure on early childhood education across Organisation for Economic Cooperation and Development (OECD) countries showed a great divergence in proportion of public spending by country.^15^ For example, 95% of the cost for early childhood education for children above the age of 3 was covered by public spending in Sweden, whereas 74% was covered by public spending in the United States in 2014. In Japan, 48% of the cost was public and 52% was private. The report also showed that Norway spent 0.9% of its gross domestic product on ECEC in 2014, whereas the United States spent 0.4% and Japan spent 0.2%. The average for the OECD countries was 0.6%.^15^

According to 2017 data, 8.9% of children at the age of 3 years, 2.7% at the age of 4 years, and 1.9% at the age of 5 years were not enrolled in center-based childcare care in Japan.^16^ However, these numbers are estimates, and their accuracy has not been verified, as they were calculated based on combined results from different national surveys conducted by different ministries with different formats and timing. There are three types of center-based childcare overseen by three different ministries in Japan: Hoiku-En by the Ministry of Health, Labour and Welfare; Youchi-En by the Ministry of Education; and Kodomo-En by the Cabinet Office.

To our knowledge, few studies have been conducted in Japan or other Asian countries to examine the relationship between socio-demographic and other factors and the use/non-use of center-based childcare. One study in Japan has shown that the proportion of the non-use of center-based childcare among children between the ages of 4 and 6 years was higher among mothers with low education/no job, as well as fathers with low income.^17^ In our study, we utilized the Longitudinal Survey of Newborns in the 21st Century, which include two nationally representative samples of families from all over Japan with rich socio-demographic information. Based on aforementioned previous findings from other developed countries and Japan, we hypothesized that parental, child, and environmental factors would influence Japanese families’ decisions over whether or not to use center-based childcare.

## METHODS

### Data source and participants

Data were drawn from the Longitudinal Survey of Newborns in the 21st Century (LSN), a national longitudinal survey of children and their families in Japan.^18^ The survey is conducted by the Japanese Ministry of Health, Labour and Welfare and is comprised of two cohorts starting in 2001 and 2010. The target populations were all infants born in Japan between January 10–17 and between July 10–17 of 2001 for the first cohort and between May 10–24 of 2010 for the second cohort. Hereafter, we refer to the two cohorts as the 2001 cohort and the 2010 cohort. Information at birth from vital statistics was linked to each child. As of 2018, the 2001 cohort has 15 waves of data, and the 2010 cohort has seven waves. For our study, we used Wave 1 to 6 from the first cohort and Wave 1 to 5 from the second cohort. The reason for using the two cohorts was to examine the consistency of results from the 2001 and 2010 cohorts. Regarding the first cohort, we only used data from infants born in July 10–17 to match the birth month to data from the second cohort (May 10–24 of 2010) because the enrollment for center-based childcare is likely to be affected by birth month. The proportion of the non-enrollment was higher among infants born in January than among those born in July probably due to “haya-umare (late born)” which refers to being born toward the end of the Japanese school year, ie, between January 1^st^ and April 1^st^. Some parents may postpone their late-born three-year-olds’ enrollment into center-based childcare until the next year because they might fear that their children experience difficulty adjusting to the classroom environment. We obtained permission from the Ministry of Health, Labour and Welfare to use the LSN data (Approval No. 0301-2), and the data up to Wave 5 were available for the 2010 cohort at the time of application.

**Figure 1.**
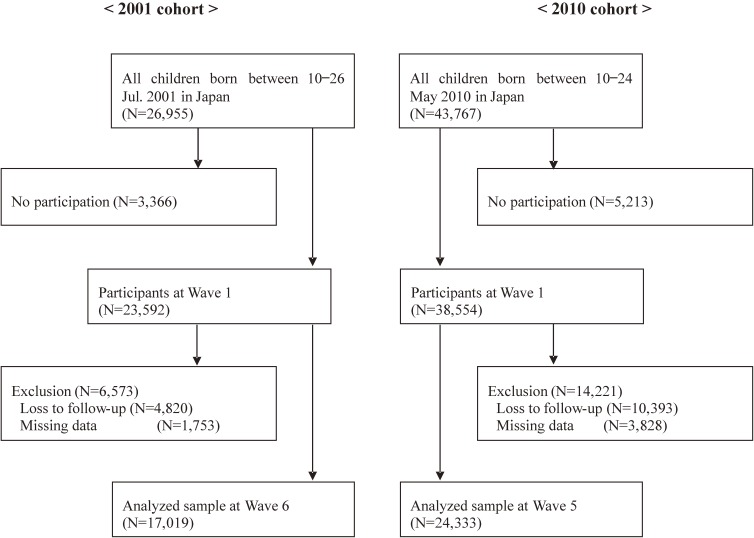
Study population

For both cohorts, the first survey was conducted when infants were 6 months old, and follow-up surveys were conducted at the following 1-year intervals: 1.5, 2.5, 3.5, 4.5, and 5.5 years. At each wave, the self-administered questionnaires were mailed to the participating families. Among the 23,592 initial participants of the 2001 cohort (response rate: 88%), 18,772 remained in the cohort at Wave 6 (retention rate: 80%). Among the 38,554 initial participants of the 2010 cohort (response rate: 88%), 28,161 remained in the cohort at Wave 5 (retention rate: 73%). After excluding participants with missing data, our analyses included 17,019 participants for the 2001 cohort and 24,333 participants for the 2010 cohort. A flow chart for describing the number of the participants is displayed as Figure 1. Compared to those who remained in the survey, those who dropped out from the survey tended to be more socially vulnerable, specifically, lower income, mothers with low education, and single-parent households, as shown in eTable 1. Ethics approval was obtained at the National Center for Child Health and Development (No. 1533).

### Variables

#### Non-use of center-based childcare

Our outcome of interest in this study was the use or non-use of center-based childcare at the ages of 3 and 4 years, assessed at Waves 5 and 6 in the 2001 cohort and at the age of 3 years, assessed at Wave 5 in the 2010 cohort. If respondents of the survey did not select childcare workers of Hoiku-En or Youchi-En (or Kodomo-En in the 2010 survey) as the primary caregiver of the child during the day on a weekday, they were considered as non-users of center-based childcare. We provide descriptions of Hoiku-En, Youchi-En, and Kodomo-En in eAppendix 1.

#### Reasons for non-use of center-based childcare

Only in the 2010 survey, respondents were asked to choose one of the following five reasons for not using childcare services such as center-based childcare: 1) No need, 2) No childcare services I want to use, 3) Needed childcare services unavailable where I live, 4) I want to use some childcare service but cannot use it due to a financial reason, and 5) other reasons. We combined options 2) and 3) into one as “accessibility reasons”.

#### Parental, child, and environmental factors

Parental factors included household income, mother’s education, mother’s employment status, household structure, number of older siblings, nationality of parents, and concerns over child rearing. Although we considered mother’s age as a candidate factor, we decided not to include it due to its high correlation with mother’s education. Child factors included sex, preterm birth, congenital diseases, and developmental delay. Environmental factors included the size of the residential area and name of the region. Full details of factor assessment are provided in eTable 2.

### Statistical analyses

We analyzed the two cohorts separately. First, we reported the baseline characteristics and proportions of non-use of center-based childcare by each characteristic when children were 3 and 4 years of age. We also examined who the responsible caregiver was for children without center-based care. Second, logistic regression analysis was conducted to estimate the crude odds ratios (cORs) and adjusted odds ratios (aORs) with 95% confidence intervals (CIs) for the non-use of center-based childcare by each parental, child, and environmental factor. In the adjusted model, we included all the factors simultaneously. Although child factors could mediate the association between parental socio-economic factors and non-use of center-based childcare, our preliminary analyses did not support a mediating role of child factors (see eTable 3). Finally, we compared the reasons for non-use of center-based childcare by the categories of factors that were significantly associated with non-use of center-based childcare in the logistic regression analyses. Analyses were conducted using SAS version 9.3 for Windows (SAS Inc., Cary, NC, USA) and STATA 13 (Stata Corp., College Station, TX, USA).

## RESULTS

### Characteristics of participants

We present the characteristics of children and their families and proportion of non-use of center-based childcare by each parental, child, and environmental factor in Table 1. The proportion of non-use of center-based child care for 3 and 4 years in the 2001 cohort was 18.0% and 5.0%, respectively, and for 3 years in the 2010 cohort it was 8.2%. This decrease in the proportion at age 3 from 18.0% in 2001 to 8.2% in 2010 may partly reflect an increase in women’s employment rate after birth, as Table 1 shows.

**Table 1.** Characteristics of children and their families and proportions of non-use of center-based childcare by birth cohort and age group

Factors	2001 cohort			2010 cohort	
	
	*N* (%)	Non-use (%)		*N* (%)	Non-use (%)
	
		3-year	4-year		3-year
All	17,019 (100.0)	18.0	5.0	24,333 (100.0)	8.2
*Parental factors*					
Income quintile					
5th (highest)	3,828 (22.5)	12.2	3.7	5,411 (22.2)	4.3
4th	3,673 (21.6)	17.9	4.7	5,396 (22.2)	6.9
3rd	3,515 (20.7)	19.5	5.3	4,630 (19.0)	8.5
2nd	3,297 (19.4)	21.0	5.3	4,853 (19.9)	10.0
1st (lowest)	2,706 (15.9)	20.3	6.7	4,043 (16.6)	12.3
Mother’s education					
4-year college or greater	2,594 (15.2)	13.4	4.9	7,125 (29.3)	6.0
Junior college	7,397 (43.5)	16.7	4.5	10,227 (42.0)	7.7
High school	6,352 (37.3)	20.5	5.4	6,136 (25.2)	11.0
Junior high school	656 (3.9)	24.2	7.3	778 (3.2)	12.7
Others	20 (0.1)	30.0	10.0	67 (0.3)	3.0
Mother’s employment status					
Not employed	11,811 (69.4)	22.7	6.3	14,076 (57.9)	11.9
Self-employed	952 (5.6)	10.6	3.6	1,029 (4.2)	5.4
Part-time employed	1,532 (9.0)	8.9	2.6	3,115 (12.8)	3.7
Full-time employed	2,664 (15.7)	4.9	1.4	6,020 (24.7)	2.4
Others	60 (0.4)	15.0	5.0	93 (0.4)	4.3
Household structure					
Two parents (two-generation)	13,237 (77.8)	18.9	5.3	20,488 (84.2)	8.2
Two parents (three-generation)	3,496 (20.5)	14.9	3.9	3,704 (15.2)	7.9
Single parent (two-generation)	120 (0.7)	10.8	4.2	45 (0.2)	11.1
Single parent (three-generation)	166 (1.0)	10.8	7.8	96 (0.4)	6.3
Number of siblings					
0	8,341 (49.0)	18.0	5.1	11,737 (48.2)	7.5
1	6,400 (37.6)	16.6	4.5	9,320 (38.3)	8.2
2	1,932 (11.4)	21.0	6.2	2,784 (11.4)	10.4
≥3	346 (2.0)	23.4	8.4	492 (2.0)	11.8
Nationality of parents					
Japanese	16,626 (97.7)	17.9	5.0	23,849 (98.0)	8.1
Foreign national	393 (2.3)	18.3	8.1	484 (2.0)	11.0
Concerns over child rearing					
Little	7,363 (43.3)	19.2	5.4	10,806 (44.4)	8.6
Some	8,857 (52.0)	17.1	4.7	12,402 (51.0)	7.7
Much	799 (4.7)	15.5	5.8	1,125 (4.6)	9.4
*Child factors*					
Child’s sex					
Boy	8,938 (52.5)	17.2	4.7	12,605 (51.8)	8.1
Girl	8,081 (47.5)	18.7	5.4	11,728 (48.2)	8.2
Preterm birth					
Full-term	16,198 (95.2)	17.8	4.8	23,101 (94.9)	8.1
Moderately preterm	735 (4.3)	19.9	8.8	1,086 (4.5)	9.3
Very preterm	86 (0.5)	20.9	9.3	146 (0.6)	17.1
Congenital diseases					
Without	16,839 (98.9)	17.9	4.4	23,865 (98.1)	8.1
With	180 (1.1)	21.7	5.0	468 (1.9)	12.0
Developmental delay					
Without	14,806 (87.0)	17.6	4.9	21,128 (86.8)	7.6
With	2,213 (13.0)	20.3	6.0	3,205 (13.2)	11.9
*Environmental factors*					
Size of residential area					
County	3,237 (19.0)	14.9	4.0	2,888 (11.9)	8.3
Small-to-medium city	10,074 (59.2)	18.9	5.2	14,609 (60.0)	8.6
Large city	3,688 (21.7)	18.1	5.5	6,740 (27.7)	7.1
Foreign country	20 (0.1)	0.0	0.0	96 (0.4)	8.3
Region					
Hokkaido	627 (3.7)	27.4	8.6	865 (3.6)	8.6
Tohoku	1,265 (7.4)	23.4	7.4	1,578 (6.5)	10.1
Kanto	5,506 (32.4)	20.7	5.5	8,246 (33.9)	8.0
Chubu	3,240 (19.0)	3.8	1.1	4,476 (18.4)	2.8
Kansai	3,069 (18.0)	24.7	6.9	4,305 (17.7)	13.6
Chugoku	1,035 (6.1)	17.6	4.8	1,460 (6.0)	9.8
Shikoku	485 (2.9)	11.6	2.7	683 (2.8)	6.4
Kyushu/Okinawa	1,772 (10.4)	18.6	5.2	2,624 (10.8)	7.1
Foreign country	20 (0.1)	0.0	0.0	96 (0.4)	8.3

Both in the 2001 and 2010 cohorts, 1–2% of children lived in a single-parent household, 2% of children had three or more siblings, and 2% were non-Japanese parents. About 5% of respondents had much concern over child rearing. About 5% of children were born preterm; 1–2% of children have been indicated as having congenital diseases; 13% of children showed some indication of developmental delay. About 30% of participants lived in the Kanto region. As we show in eTable 4, about 90% of children who were not enrolled in center-based childcare were cared for by their mothers.

### Factors associated with non-use of center-based childcare

In Table 2, we present associations between each parental, child, and environmental factor and the non-use of center-based childcare, adjusting for all the factors simultaneously. Crude odds ratios are shown in eTable 5. In the 2001 cohort, the general tendency of associations remained consistent with age progression, though the strength of the associations varied. Because results were similar between ages, we mainly described results at age 4.

**Table 2.** Adjusted odds ratios with 95% confidence intervals for the associations between parental, child, and environmental factors and non-use of center-based childcare with the 2001 and 2010 cohorts and age group

	2001 cohort				2010 cohort	
	
	3-year		4-year		3-year	
	aOR (95% CI)		aOR (95% CI)		aOR (95% CI)	
*Parental factors*						
Income quintile						
5th (highest)	Ref		Ref		Ref	
4th	1.27	(1.11, 1.46)^*^	1.09	(0.86, 1.38)	1.25	(1.05, 1.49)^*^
3rd	1.34	(1.16, 1.53)^*^	1.19	(0.94, 1.51)	1.37	(1.14, 1.63)^*^
2nd	1.45	(1.26, 1.66)^*^	1.18	(0.92, 1.50)	1.58	(1.33, 1.88)^*^
1st (lowest)	1.45	(1.25, 1.69)^*^	1.54	(1.20, 1.98)^*^	1.92	(1.60, 2.30)^*^
Mother’s education						
4-year college or greater	0.85	(0.74, 0.97)^*^	1.21	(0.98, 1.50)	0.90	(0.79, 1.03)
Junior college	Ref		Ref		Ref	
High school	1.21	(1.11, 1.33)^*^	1.09	(0.93, 1.28)	1.31	(1.17, 1.47)^*^
Junior high school	1.49	(1.22, 1.83)^*^	1.36	(0.98, 1.89)	1.37	(1.08, 1.73)^*^
Others	2.35	(0.85, 6.51)	2.21	(0.49, 9.96)	0.38	(0.09, 1.59)
Mother’s employment status						
Not employed	Ref		Ref		Ref	
Self-employed	0.40	(0.32, 0.50)^*^	0.54	(0.38, 0.77)^*^	0.41	(0.31, 0.54)^*^
Part-time employed	0.31	(0.26, 0.37)^*^	0.36	(0.26, 0.51)^*^	0.26	(0.22, 0.32)^*^
Full-time employed	0.20	(0.17, 0.24)^*^	0.24	(0.17, 0.33)^*^	0.23	(0.20, 0.28)^*^
Others	0.68	(0.33, 1.42)	0.84	(0.26, 2.71)	0.36	(0.13, 0.99)^*^
Household structure						
Two parents (two-generation)	Ref		Ref		Ref	
Two parents (three-generation)	0.87	(0.78, 0.97)^*^	0.81	(0.67, 1.00)	0.97	(0.85, 1.12)
Single parent (two-generation)	0.55	(0.30, 1.03)	0.56	(0.22, 1.45)	1.31	(0.49, 3.47)
Single parent (three-generation)	0.64	(0.38, 1.08)	1.43	(0.77, 2.68)	0.70	(0.30, 1.64)
Number of siblings						
0	Ref		Ref		Ref	
1	0.87	(0.80, 0.96)^*^	0.89	(0.76, 1.05)	1.05	(0.95, 1.17)
2	1.20	(1.06, 1.37)^*^	1.28	(1.03, 1.59)^*^	1.42	(1.22, 1.64)^*^
≥3	1.53	(1.17, 2.02)^*^	1.92	(1.28, 2.89)^*^	1.59	(1.18, 2.15)^*^
Nationality of parents						
Japanese	Ref		Ref		Ref	
Foreign national	1.06	(0.80, 1.41)	1.48	(1.00, 2.24)^*^	1.35	(1.00, 1.83)^*^
Concerns over child rearing						
Little	Ref		Ref		Ref	
Some	0.85	(0.78, 0.92)^*^	0.85	(0.74, 1.00)	0.89	(0.81, 0.98)^*^
Much	0.71	(0.58, 0.88)^*^	1.00	(0.72, 1.38)	1.02	(0.81, 1.27)
*Child factors*						
Child’s sex						
Boy	Ref		Ref		Ref	
Girl	1.12	(1.03, 1.22)^*^	1.18	(1.03, 1.36)^*^	1.06	(0.96, 1.16)
Preterm birth						
Full-term	Ref		Ref		Ref	
Moderately preterm	1.17	(0.96, 1.42)	1.97	(1.50, 2.59)^*^	1.15	(0.93, 1.43)
Very preterm	1.21	(0.70, 2.09)	1.86	(0.88, 3.94)	1.84	(1.16, 2.92)^*^
Congenital diseases						
Without	Ref		Ref		Ref	
With	1.55	(1.05, 2.27)^*^	0.92	(0.45, 1.91)	1.40	(1.04, 1.89)^*^
Developmental delay						
Without	Ref		Ref		Ref	
With	1.08	(0.95, 1.21)	1.12	(0.91, 1.36)	1.37	(1.20, 1.55)^*^
*Environmental factors*						
Size of residential area						
County	Ref		Ref		Ref	
Small-to-medium city	1.23	(1.09, 1.38)^*^	1.25	(1.02, 1.54)^*^	1.07	(0.92, 1.24)
Large city	0.98	(0.85, 1.13)	1.14	(0.89, 1.45)	0.90	(0.76, 1.07)
Foreign country	—	—	—	—	1.05	(0.49, 2.23)
Region						
Hokkaido	1.28	(1.05, 1.55)^*^	1.52	(1.11, 2.07)^*^	0.88	(0.68, 1.13)
Tohoku	1.35	(1.15, 1.58)^*^	1.61	(1.25, 2.07)^*^	1.21	(1.00, 1.46)^*^
Kanto	Ref		Ref		Ref	
Chubu	0.14	(0.12, 0.18)^*^	0.21	(0.15, 0.29)^*^	0.29	(0.23, 0.35)^*^
Kansai	1.19	(1.06, 1.32)^*^	1.23	(1.02, 1.48)^*^	1.64	(1.45, 1.85)^*^
Chugoku	0.83	(0.70, 0.99)^*^	0.92	(0.67, 1.26)	1.21	(0.99, 1.47)
Shikoku	0.49	(0.36, 0.66)^*^	0.47	(0.26, 0.82)^*^	0.70	(0.51, 0.97)^*^
Kyushu/Okinawa	0.86	(0.75, 0.99)^*^	0.94	(0.73, 1.20)	0.80	(0.67, 0.95)^*^
Foreign country	—	—	—	—	—	—

aOR, adjusted odds ratio; CI, confidence interval.

The significant parental factors for non-use of center-based childcare included lower household income, mother’s low education, mother’s employment status, more siblings, and non-Japanese parents. Lower-income families were more likely to choose non-use of center-based childcare compared with those from the highest-income families (ie, the fifth quintile). The aOR for the first quintile was 1.54 (95% CI, 1.20–1.98). Mothers with only junior high school education were more likely to choose non-use of center-based childcare than those mothers who have Junior college education (aOR 1.36; 95% CI, 0.98–1.89). As expected, when mothers engaged in some form of work, children were more likely to be enrolled in center-based childcare. Families with more children were more likely to choose non-use of center-based childcare compared to those with one child; the aOR was 1.28 (95% CI, 1.03–1.59) for two siblings and 1.92 (95% CI, 1.28–2.89) for three or more siblings. We did not observe any consistent pattern with household structure or mother’s concern over child rearing.

The significant child factor for non-use of center-based childcare was preterm birth. Children who were born moderately preterm may be more likely to not receive center-based childcare than those who were born full-term (aOR 1.97; 95% CI, 1.50–2.59).

The significant environmental factors for non-use of center-based childcare included small-to-medium city and some regions. Children who lived in small-to-medium cities were more likely to not receive center-based childcare compared to those who lived in county areas (aOR 1.25; 95% CI, 1.02–1.54). Regarding the regions, children who lived in Hokkaido, Tohoku, and Kansai were more likely and children who lived in Chubu and Shikoku were less likely to not receive center-based childcare compared to those who lived in Kanto.

Similar associations were observed in the 2010 cohort. The significant parental, child, and environmental factors for center-based childcare non-use included lower household income, mother’s low education, mother’s employment status, more siblings, parents of foreign nationality, preterm birth, and some regions. Associations of congenital diseases and developmental delay with non-use of center-based childcare were also observed in the 2010 cohort.

### Reasons for non-use of center-based childcare

In addition to the factors associated with non-use of center-based childcare, we explored the reasons for non-use of center-based childcare with the 2010 cohort. As we show in Table 3, socially vulnerable families with low income, mother’s low education, mother’s employment, and parents of foreign nationality may not to be able to use center-based childcare due to financial reasons. For example, higher percentages of the lowest income families that did not use center-based childcare chose “financial reasons” compared to the highest income families (13.8% for the first quintile vs 4.6% for the fifth quintile). The percentage of “no need” was higher among families with three or more siblings (75.0% compared to 68.4% for families with no siblings). Families of children with some developmental or health problems may choose non-use of childcare, possibly due to accessibility issues. For example, the percentage of “accessibility reasons” was higher among children born very preterm compared with children born full-term (23.5% vs 9.4%). The percentages were also higher among children with congenital disease or developmental delay compared to those without (10.4% vs 9.5% for congenital disease and 11.0% vs 9.2% for developmental delay).

**Table 3.** Reasons for non-use of center-based childcare in the 2010 cohort

	No need	Financial reasons	Accessibility reasons	Others
All	70.1	9.6	9.5	10.7
Income quintile				
5th (highest)	74.4	4.6	7.4	13.6
4th	71.0	7.0	9.8	12.2
3rd	74.5	7.1	8.2	10.2
2nd	69.8	11.6	8.8	9.7
1st (lowest)	64.4	13.8	12.0	9.9
Mother’s education				
4-year college or greater	76.3	4.3	10.2	9.2
Junior college	73.1	8.0	8.6	10.2
High school	64.2	13.2	10.3	12.3
Junior high school	58.5	22.0	8.5	11.0
Others	100.0	0.0	0.0	0.0
Mother’s employment status				
Not employed	72.2	9.4	8.7	9.7
Self-employed	56.5	4.4	23.9	15.2
Part-time employed	50.0	20.5	17.1	12.5
Full-time employed	63.0	6.5	8.3	22.2
Others	75.0	0.0	25.0	0.0
Number of siblings				
0	68.4	8.1	11.3	12.2
1	71.6	10.7	7.7	10.0
2	70.1	11.0	9.5	9.5
≥3	75.0	11.5	7.7	5.8
Nationality of parents				
Japanese	70.6	9.5	9.3	10.6
Foreign national	50.0	15.0	17.5	17.5
Preterm birth				
Full-term	70.3	9.6	9.4	10.7
Moderately preterm	71.8	9.4	8.2	10.6
Very preterm	41.2	17.7	23.5	17.7
Congenital diseases				
Without	70.3	9.7	9.5	10.5
With	62.5	8.3	10.4	18.8
Developmental delay				
Without	71.5	9.5	9.2	9.8
With	63.8	10.4	11.0	14.8

Note: Values are percentages.

## DISCUSSION

### Main findings

We found that children from socially disadvantaged families, characterized by low household income, mother’s low education, non-Japanese parent, and more siblings, were less likely to use center-based childcare despite the potential benefits of utilizing center-based childcare, such as reduction of behavior problems.^7^ We also found that children with health and developmental problems may be less likely to receive center-based childcare. These findings suggest that parental decision over whether or not to use center-based childcare may depend on the families’ economic and other difficulties. The utilization of two nationally representative samples of children from all over Japan and consistent findings from the two cohorts strengthen the validity of our findings.

### Comparisons with previous studies

Our results are consistent with the findings of previous studies conducted in the United State, Canada, Australia, Japan, and other countries. The enrollment rates of ECEC for children between the ages of 3 and 5 in developed countries range from 66% in the United States to 100% in France in 2016, with the average of 86% among OECD countries.^15^ Despite the variability in enrollment rates and cultural context, previous studies seem to indicate that Julian Tudor Hart’s inverse care law, meaning that needy families have least access to services, such as good medical care, may be operating with enrollment in early childhood education in developed countries.^19^^,^^20^ Those studies indicated that socio-economic disadvantages, including lower household income, mother’s lower educational attainments, mother’s unemployment, many children in household, and non-native parents, were associated with inequality in enrolling in ECEC.^9^^–^^14^ In addition, our findings suggested children’s health and developmental issues as a potential barrier against the use of ECEC among Japanese families. In previous studies, two studies conducted in the United States and Norway considered child health indicators (preterm birth, low birth weight, and illness), but no association between these indicators and the selection of ECEC was observed.^9^^,^^14^ Three studies reported inconsistent results on the association between developmental status and the selection of ECEC.^9^^,^^10^^,^^14^ Two studies conducted in the United States and Norway found no association,^9^^,^^14^ but one study conducted in the United States found a positive association between low cognitive development and the selection of ECEC.^10^

### Potential mechanisms

We provide several explanations to link socio-economic disadvantages and non-use of center-based childcare among Japanese children in the 2001 and 2010 cohorts. First, our results suggest that non-use of center-based childcare can be attributed to financial reasons. Odds ratios in Table 2 and percentages in Table 3 show a linear relationship between higher proportions of non-use of center-based childcare and non-use due to financial reasons and lower household income. However, because the childcare fee for Hoiku-En is determined according to household income, a lower fee for lower-income families should reduce the enrollment gap. One possible explanation for the discrepancy is the burden of additional payments, such as fees for extracurricular activities, monthly lunch fee, and fee for extending childcare hours.^21^ Lower household income may also be a marker for some other issues, such as mental health problems among parents. Parents with poor mental health status may experience difficulty performing daily routines, such as sending children to and picking children up from childcare centers. A community survey conducted in an impoverished area in Tokyo showed that poor mental health status was prevalent among socio-economically deprived families.^22^ Our results also suggest that non-use of center-based childcare can be strongly attributed to mother’s unemployment. This may be due to at least two reasons. According to the Japanese Child Welfare Act, parental employment is one of the conditions for enrolling their children in Hoiku-en. In addition, low-income families with unemployed mothers may not be able to afford to pay for Youchi-En because Youchi-En tends to cost more than Hoiku-En, with the absence of sliding-scale enrollment. When there are multiple children of varying ages in a household, parents may expect an older brother or sister to take care of their younger siblings and try to reduce the cost of childcare. Consequently, the parents may not feel the need to send their children to center-based childcare, as the higher percentage of “no need” in Table 3 indicates.

Children’s health and developmental problems also may become a barrier for parents to use center-based childcare. Preterm birth and congenital diseases may be directly associated with the non-use of ECEC and indirectly associated through mediating developmental delay. For example, being born at 32 gestational weeks or earlier, these children are at risk of having some health problems, including cerebral palsy, home oxygen therapy use, visual impairment, and cognitive impairment.^23^ Most Japanese childcare centers do not have the capacity to accommodate the medical needs of such children due to the absence of specially trained staff (such as nurses), which results in refusal of enrollment. Reasons for non-use, presented in Table 3, seem to be in accordance with this interpretation. The proportions of non-use of center-based childcare due to financial, accessibility, and other reasons were much higher for parents with children born very preterm compared to those with children born full-term or moderately preterm. Raising children with special health care needs places extra financial and physical burden on parents; therefore, lack of public support for these parents has become an issue in Japanese society in recent years.^24^ High percentages of “others” reasons for non-use of center-based childcare among those with congenital disease and developmental delay compared to those without may reflect the same incapacity issue among Japanese childcare facilities.

Higher likelihood of choosing non-use of center-based childcare among non-Japanese parents compared to Japanese parents may reflect their cultural preference over childrearing, linguistic and financial barriers, or unknown reasons. Despite the increasing number of residents with foreign nationality in Japan, studies on how they raise children in Japan remain scarce.^25^ To build on findings of this study and extend further, we would need to conduct more complex statistical analyses, such as structural equation modeling, to consider the temporal sequence of events and explore causal relationships.

### Strengths and limitations

This study’s strengths include LSN’s large nationally representative sample, linked birth data, use of two cohorts from 2001 and 2010, and being the first study to analyze the determinants of selection of center-based childcare in Asian countries. This study also has a few limitations. First, the assessment of outcomes and some parental and child’s factors were based on parental self-report. Thus, we cannot eliminate the possibility of misclassification, which might bias the estimates. Second, the strength of associations may be underestimated because of the higher rate of loss to follow-up among socially disadvantaged families. Third, we lacked data on other factors, such as child temperament and maternal depression, that may be associated with selection of center-based childcare. Finally, we could not examine the consistency of results at age 4 between the 2001 and 2010 cohorts because of the unavailability of the 2010 data.

### Policy implications

Our findings have policy implications applicable both to the local context of Japan and to the context of other developed countries. First, the central government should install a surveillance system to monitor the prevalence of non-users of center-based childcare. Currently, the central government and municipalities do not have the systems in place to collect reliable data because of the complexities of the Japanese early childhood education system. Second, public support for vulnerable families beginning in pregnancy and following through the early years up to the enrollment into elementary school needs improvement. Home visits to non-users of center-based childcare by a government agency may be necessary because our results suggest these families are likely to be socially vulnerable and isolated and thus have high needs for support. Third, the number of childcare centers that provide appropriate care to children with special health needs to be increased. Currently, the families, assumingly mothers in most cases, are required to take care of the children with special health and developmental needs at home, as the early years are crucial for promoting children’s cognitive, socio-emotional, and physical development.^26^ Finally, because the rate of children whose father or mother is non-Japanese has been gradually increasing from 1.7% in 1990 to 3.7% in 2016,^27^ more research and policy attention should be given to non-utilization of ECEC among such children.

### Conclusions

Socio-economically disadvantaged children are less likely to receive ECEC than socio-economically advantaged children. Children with health and developmental issues also showed a decreased likelihood of using ECEC compared to optimally developing children. Policy makers in Japan should redress the access gap in center-based childcare among Japanese families to alleviate future socio-economic inequalities.

## APPENDIX A. SUPPLEMENTARY DATA

eTable 1. Comparison of the participants who were and were not followed at Wave 6 (2001 cohort) or Wave 5 (2010 cohort) by birth cohort

eTable 2. Details of factor assessment

eTable 3. Associations between parental and child factors

eTable 4. Responsible caregiver for children without center-based childcare by birth cohort and age group

eTable 5. Crude odds ratios with 95% confidence intervals for the associations between parental, child, and environmental factors and non-use of center-based childcare with the 2001 and 2010 cohorts and age group

eAppendix 1. Three types of center-based childcare in Japan
