# Supplementary material for: Socio-Economic Disparities in Early Childhood Education Enrollment: Japanese Population-Based Study
Source: J Epidemiol. 2020 Mar 5;30(3):143–50. doi: 10.2188/jea.JE20180216 (PMC7025920; doi:10.2188/jea.JE20180216)
Supplement: Supplementary file 1 [file je-30-143-s001.pdf]

eTable 1. Comparison of the participants who were and were not followed at Wave 6 (2001 cohort) or Wave 5 (2010 cohort) by birth cohort

Baseline characteristics [a]	2001 cohort, N (%)					2010 cohort, N (%)				
	Not included (N=6,573)		Included (N=17,019)		*P*-value [b]	Not included (N=14,221)		Included (N=24,333)		*P*-value [b]
Parental factors										
Income quintile										
5th (highest)	732	(14.6)	3828	(22.5)	< 0.01	1,633	(15.3)	5,411	(22.2)	< 0.01
4th	823	(16.4)	3673	(21.6)		1,860	(17.5)	5,396	(22.2)	
3rd	897	(17.8)	3515	(20.7)		1,859	(17.5)	4,630	(19.0)	
2nd	1,114	(22.2)	3297	(19.4)		2,425	(22.8)	4,853	(19.9)	
1st (lowest)	1,463	(29.1)	2706	(15.9)		2,862	(26.9)	4,043	(16.6)	
Missing	1,544					3,582				
Mother's education										
4-year college or greater	462	(9.4)	2,594	(15.2)	< 0.01	1,663	(18.6)	7,125	(29.3)	< 0.01
Junior college	1,738	(35.4)	7,397	(43.5)		3,460	(38.7)	10,227	(42.0)	
High school	2,213	(45.1)	6,352	(37.3)		2,982	(33.3)	6,136	(25.2)	
Junior high school	485	(9.9)	656	(3.9)		812	(9.1)	778	(3.2)	
Others	9	(.2)	20	(.1)		27	(0.3)	67	(0.3)	
Missing	1,666					5,277				
Household structure										
Two parents (two-generation)	4,886	(74.3)	13,237	(77.8)	< 0.01	11,056	(77.8)	20,488	(84.2)	< 0.01
Two parents (three-generation)	1,387	(21.1)	3,496	(20.5)		2,351	(16.5)	3,704	(15.2)	
Single parent (two-generation)	133	(2.0)	120	(.7)		342	(2.4)	45	(0.2)	
Single parent (three-generation)	167	(2.5)	166	(1.0)		466	(3.3)	96	(0.4)	
Missing	0					6				
Number of siblings										
0	3,292	(50.1)	8,341	(49.0)	< 0.01	6,781	(47.7)	11,737	(48.2)	< 0.01
1	2,358	(35.9)	6,400	(37.6)		5,050	(35.5)	9,320	(38.3)	
2	726	(11.0)	1,932	(11.4)		1,899	(13.4)	2,784	(11.4)	
≥3	197	(3.0)	346	(2.0)		485	(3.4)	492	(2.0)	
Missing	0					6				
Nationality of parents										
Japanese	6,138	(93.4)	16,626	(97.7)	< 0.01	13,157	(92.5)	23,849	(98.0)	< 0.01
Foreign national	435	(6.6)	393	(2.3)		1,064	(7.5)	484	(2.0)	

Mother's age										
<20	155	(2.4)	81	(.5)	< 0.01	278	(2.0)	58	(0.2)	< 0.01
20–39	6,316	(96.1)	16,615	(97.6)		13,540	(95.2)	23,380	(96.1)	
≥40	102	(1.6)	323	(1.9)		403	(2.8)	895	(3.7)	
Child factors										
Child's sex										
Boy	3,427	(52.1)	8,938	(52.5)	0.60	7,239	(50.9)	12,605	(51.8)	0.09
Girl	3,146	(47.9)	8,081	(47.5)		6,982	(49.1)	11,728	(48.2)	
Preterm birth										
Full-term	6,179	(94.2)	16198	(95.2)	< 0.01	13,348	(93.9)	23,101	(94.9)	< 0.01
Moderately preterm	333	(5.1)	735	(4.3)		770	(5.4)	1,086	(4.5)	
Very preterm	44	(.7)	86	(.5)		96	(0.7)	146	(0.6)	
Missing	17					7				
Congenital diseases										
With	38	(.8)	180	(1.1)	< 0.01	168	(1.2)	468	(1.9)	< 0.01
Without	4,945	(99.2)	16,839	(98.9)		14,053	(98.8)	23,865	(98.1)	
Missing	1,590					0				

[a] These include factors that was assessed at the first wave except for the mother's education (assessed at Wave 2).

[b] Chi-square test was conducted to compare the participants who were and were not followed by the categories of factors.

eTable 2. Details of factor assessment

Factor	Data source	Details of assessment and categorization
Parental factors		
Household income	Wave 1	Household income was categorized into quintiles.
Mother's education	Wave 2	Educational level was subdivided into graduation from junior high school, high school, junior college, college or greater, and others.
Mother's employment status	Wave 2	Mother's employment status was categorized into not employed, self-employed, part-time employed, full-time employed, and others.
Household structure	Wave 1	Household structure was categorized into the following four groups, considering the presence of grandparents who might offer childcare support: two parents (two-generation), two parents (three-generation), single parent (two-generation), and single parent (three-generation).
Number of siblings	Wave 1	The number of siblings was categorized into 0, 1, 2, and ≥3.
Nationality of parents	Birth records	Nationality of parents was determined based on how fathers or mothers listed their nationality in the birth record, i.e., Japanese or citizens of countries other than Japan.
Concerns over child rearing	Wave 2	Concerns over child rearing were assessed at three levels, i.e., little, some, and much.
Child factors		
Sex	Birth records	Sex was assigned as boy or girl.
Preterm birth	Birth records	Preterm birth was defined as delivery before 37 gestational weeks and was further categorized into very preterm (i.e., 31 weeks or earlier) and moderately preterm (i.e., 32–36 weeks).
Congenital diseases	Wave 2	Congenital diseases were assessed based on parental report of visiting a physician for congenital diseases during the previous year.
Developmental delay	Wave 3	Developmental delay was defined as children whom their parents reported they could not do at least one of the following six activities: 1) walk, 2) run, 3) climb stairs, 4) say meaningful words such as "mama" and "bubu" (meaning a toy car in Japanese), 5) say a two-word sentence, and 6) say one's name.
Environmental factors		

Size of residential area	Wave 3	The size of the residential area was classified into three levels, i.e., counties, small-to-medium cities, and large cities, and an additional category, namely, foreign country.
Region in which survey participants were residing	Wave 3	Forty-seven prefectures were grouped into eight regions, i.e., Hokkaido, Tohoku, Kanto, Chubu, Kansai, Chugoku, Shikoku, and Kyushu/Okinawa, and an additional category, namely, foreign country.

eTable 3. Associations between parental and child factors

	Child factors							
	2001 birth cohort				2010 birth cohort			
	Preterm birth		Congenital	Developmental	Preterm birth		Congenital	Developmental
Parental factors	Very	Moderately	diseases	delay	Very	Moderately	diseases	delay
Income quintile								
5th (highest)	0.4	4.1	1.4	12.8	0.6	4.6	2.1	10.9
4th	0.3	4.0	1.3	13.2	0.5	4.8	2.2	13.6
3rd	0.6	4.5	0.9	14.0	0.6	4.7	1.9	14.3
2nd	0.5	4.3	0.8	11.5	0.7	4.1	1.7	13.5
1st (lowest)	0.8	4.9	0.7	13.5	0.6	3.9	1.6	14.1
Chi-square test	p=0.09		p=0.01	p=0.03	p=0.27		p=0.16	p < 0.01
Mother's education								
4-year college or greater	0.6	3.8	1.4	12.9	0.5	4.3	1.9	11.8
Junior college	0.5	4.2	1.2	12.3	0.6	4.7	1.9	13.2
High school	0.4	4.7	0.8	13.8	0.7	4.3	2.0	14.8
Junior high school	0.8	4.3	0.9	14.0	0.9	5.0	1.4	13.9
Others	0.0	10.0	0.0	0.0	0.0	0.0	3.0	6.0
Chi-square test	p=0.49		p=0.08	p=0.03	p=0.34		p=0.81	p < 0.01

Note: Numbers are percentages.

eTable 4. Responsible caregiver for children without center-based childcare by birth cohort and age group

	2001 cohort		2010 cohort
	3-year	4-year	3-year
Mother	92.7	90.2	95.6
Father	0.1	0.5	0.6
Grandmother (maternal)	2.4	1.9	2.1
Grandfather (maternal)	0.2	0.2	0.1
Grandmother (paternal)	2.0	2.6	1.2
Grandfather (paternal)	0.1	0.2	0.1
Others	2.5	4.4	0.4

Note: Numbers are percentages.

eTable 5. Crude odds ratios with 95% confidence intervals for the associations between parental, child, and environmental factors and non-use of center-based childcare with the 2001 and 2010 cohorts and age group

	2001 cohort				2010 cohort	
	3-year		4-year		3-year	
	cOR (95% CI)		cOR (95% CI)		cOR (95% CI)	
Parental factors						
Income quintile						
5th (highest)	Ref		Ref		Ref	
4th	1.56	(1.38, 1.78)*	1.28	(1.02, 1.60)*	1.66	(1.40, 1.96)*
3rd	1.74	(1.54, 1.98)*	1.46	(1.17, 1.82)*	2.08	(1.76, 2.46)*
2nd	1.91	(1.68, 2.17)*	1.44	(1.15, 1.80)*	2.48	(2.11, 2.92)*
1st (lowest)	1.82	(1.59, 2.09)*	1.86	(1.49, 2.33)*	3.14	(2.67, 3.69)*
Mother's education						
4-year college or greater	0.77	(0.82, 5.58)*	1.09	(0.89, 1.35)	0.76	(0.67, 0.86)*
Junior college	Ref		Ref		Ref	
High school	1.29	(1.19, 1.41)*	1.20	(1.03, 1.40)*	1.49	(1.33, 1.66)*
Junior high school	1.60	(1.32, 1.93)*	1.66	(1.22, 2.28)*	1.75	(1.40, 2.19)*
Others	2.14	(0.82, 5.58)*	2.34	(0.54, 10.14)	0.37	(0.09, 1.52)
Mother's employment status						
Not employed	Ref		Ref		Ref	
Self-employed	0.41	(0.33, 0.50)*	0.55	(0.39, 0.79)*	0.43	(0.33, 0.56)*
Part-time employed	0.33	(0.28, 0.40)*	0.39	(0.28, 0.54)*	0.28	(0.23, 0.34)*
Full-time employed	0.18	(0.15, 0.21)*	0.22	(0.16, 0.30)*	0.18	(0.15, 0.22)*
Others	0.60	(0.30, 1.23)	0.79	(0.25, 2.52)	0.33	(0.12, 0.91)*
Household structure						
Two parents (two-generation)	Ref		Ref		Ref	
Two parents (three-generation)	0.75	(0.68, 0.83)*	0.72	(0.59, 0.87)*	0.96	(0.84, 1.09)
Single parent (two-generation)	0.52	(0.29, 0.93)*	0.78	(0.32, 1.91)	1.40	(0.55, 3.55)
Single parent (three-generation)	0.52	(0.32, 0.85)*	1.52	(0.86, 2.69)	0.75	(0.33, 1.71)
Number of siblings						
0	Ref		Ref		Ref	
1	0.90	(0.83, 0.98)*	0.88	(0.76, 1.03)	1.11	(1.00, 1.23)
2	1.21	(1.07, 1.37)*	1.24	(1.00, 1.52)*	1.44	(1.25, 1.66)*
≥3	1.39	(1.08, 1.79)*	1.72	(1.16, 2.55)*	1.66	(1.25, 2.20)*
Nationality of parents						
Japanese	Ref		Ref		Ref	
Foreign national	1.03	(0.79, 1.33)	1.70	(1.18, 2.46)*	1.40	(1.05, 1.86)*
Concerns over child rearing						
Little	Ref		Ref		Ref	
Some	0.87	(0.81, 0.95)*	0.86	(0.74, 0.99)*	0.89	(0.81, 0.98)*
Much	0.78	(0.63, 0.95)*	1.07	(0.78, 1.47)	1.11	(0.90, 1.37)
Child factors						
Child's sex						
Boy	Ref		Ref		Ref	
Girl	1.11	(1.02, 1.20)*	1.15	(1.00, 1.32)*	1.02	(0.93, 1.12)
Preterm birth						
Full-term	Ref		Ref		Ref	
Moderately preterm	1.14	(0.95, 1.38)	1.91	(1.47, 2.49)*	1.17	(0.95, 1.45)

Very preterm	1.22	(0.73, 2.05)	2.02	(0.97, 4.20)	2.36	(1.53, 3.64)*
Congenital diseases						
Without	Ref		Ref		Ref	
With	1.27	(0.89, 1.81)	0.88	(0.43, 1.79)	1.55	(1.17, 2.05)*
Developmental delay						
Without	Ref		Ref		Ref	
With	1.19	(1.07, 1.34)*	1.25	(1.03, 1.51)*	1.64	(1.46, 1.85)*
Environmental factors						
Size of residential area						
County	Ref		Ref		Ref	
Small-to-medium city	1.34	(1.20, 1.49)*	1.34	(1.10, 1.63)*	1.04	(0.90, 1.21)
Large city	1.26	(1.11, 1.44)*	1.40	(1.12, 1.76)*	0.85	(0.72, 0.99)*
Foreign country	—	—	—	—	1.01	(0.48, 2.10)
Region						
Hokkaido	1.45	(1.21, 1.75)*	1.62	(1.20, 2.19)*	1.07	(0.83, 1.38)
Tohoku	1.17	(1.02, 1.36)*	1.36	(1.07, 1.73)*	1.28	(1.07, 1.54)*
Kanto	Ref		Ref		Ref	
Chubu	0.15	(0.13, 0.18)*	0.20	(0.14, 0.28)*	0.32	(0.27, 0.39)*
Kansai	1.26	(1.13, 1.40)*	1.28	(1.07, 1.54)*	1.80	(1.60, 2.03)*
Chugoku	0.82	(0.69, 0.98)*	0.87	(0.64, 1.19)	1.24	(1.03, 1.50)*
Shikoku	0.50	(0.38, 0.67)*	0.47	(0.27, 0.83)*	0.79	(0.58, 1.08)
Kyushu/Okinawa	0.88	(0.76, 1.00)	0.94	(0.74, 1.20)	0.88	(0.74, 1.04)
Foreign country	—	—	—	—	1.04	(0.50, 2.16)

CI, confidence interval; cOR, crude odds ratio.

eAppendix 1. Three types of center-based childcare in Japan

The Japanese system of early childhood education and care (ECEC) and the cultural context are different from those in the European countries and the United States. In Japan, two types of center-based ECEC programs have been institutionalized by the government and have been the main source of non-parental childcare. These programs receive public funding if facilities fulfill the standards set by the national and local governments. The first type, known as Hoiku-En, is administered under the Ministry of Health, Labour and Welfare, and a full-day program is provided for children between the ages of 0 and 6. Because this type is considered to be a welfare program for working parents, the fee is on a sliding scale according to their household income, meaning lower fees for families with lower income. Families that receive public assistance can enroll their children in the program for free and are often given a high priority for enrollment. The second type, known as Youchi-En, is administered under the Ministry of Education, Culture, Sports, Science and Technology, and a half-day program is provided for children between the ages of 3 and 6. The third type, known as Kodomo-En, was institutionalized in 2006 and is administered under the Cabinet Office. This type is a hybrid of the first two types and provides a full-day or half-day program for children between the ages of 0 and 6.

Because the programs need to comply with standards set by the government in order to receive public funding, the minimum quality of education and care is likely to be maintained among licensed facilities though great variability in quality is most likely to be present. Although the availability of center-based childcare has been a problem for working mothers in metropolitan areas such as Tokyo, the issue of short supply has been limited to

children under the age of 2 because of the teacher-child ratio: 6 or fewer children for one teacher for the child's age of 2 and 20 or more children for one teacher for the child's age of 3 or above. In other words, the availability of center-based childcare for children above the age of 3 years is not likely to be a problem. Other types of ECEC in Japan, such as childcare for a small number of children by a professional childminder/baby-sitter at one's home, have been rare in Japan.
